# Effect of *Koji* on Flavor Compounds and Sensory Characteristics of Rice *Shochu*

**DOI:** 10.3390/molecules28062708

**Published:** 2023-03-16

**Authors:** Huawei Yuan, Li Tan, Yu Zhao, Yuting Wang, Jianlong Li, Guangqian Liu, Chao Zhang, Kunyi Liu, Songtao Wang, Kai Lou

**Affiliations:** 1 Solid-State Fermentation Resource Utilization Key Laboratory of Sichuan Province, Faculty of Quality Management and Inspection Quarantine, Yibin University, Yibin 644000, China; 2 College of Food Science, Sichuan Agricultural University, Ya’an 625014, China; 3 Luzhou Laojiao Co., Ltd., Luzhou Pinchuang Technology Co., Ltd., National Engineering Technology Research Center of Solid-State Brewing, Luzhou 646000, China; 4 College of Wuliangye Technology and Food Engineering, Yibin Vocational and Technical College, Yibin 644003, China

**Keywords:** *koji*, rice *shochu*, sensory characteristics, fermentation, aroma characterization

## Abstract

*Koji* is an important starter for rice *shochu* brewing and influences the rice *shochu* quality. Consequently, we studied the impacts of *koji* on the flavor compounds and sensory characteristics of rice *shochu* using molds *Aspergillus kawachii* SICC 3.917 (A-K), *Aspergillus oryzae* SICC 3.79(A-O), *Aspergillus Niger* CICC 2372 (A-N), *Rhizopus oryzae* CICC 40260 (R-O), and the traditional starter Qu (control). The effects of *koji* on the aroma components, free amino acids (FAAs), and overall sensory aspects of rice *shochu* were studied. These findings indicated that *koji* significantly affected the rice *shochu*’s quality. The content of total FAAs in rice *shochu* A-K (30.586 ± 0.944 mg/L) and A-O (29.919 ± 0.278 mg/L) was higher than others. The content of flavor compounds revealed that the aroma of rice *shochu* with various *koji* varied greatly from the smells of alcohols and esters. *Shochu* A-O had a higher concentration of aroma compounds and it exhibited a strong aroma and harmonious taste compared with the others. This research using taste compounds, FAAs, flavor intensity, and partial least squares regression (PLSR) showed that *shochu* A-O appeared to possess the best sensory qualities, with elevated concentrations of alcohols and sweet FAAs and lesser concentrations of sour FAAs. Therefore, the A-O mold is promising for the manufacture of rice *shochu* with excellent flavor and sensory characteristics.

## 1. Introduction

Rice *shochu* is an alcoholic beverage made from rice using fermentation starters including *koji* and *Saccharomyces cerevisiae* [1]. Rice *shochu* brewing is a typical submerged fermentation process primarily composed of a *koji* starting culture in a fairly sealed tank, similar to rice wine. The starter is critical for rice *shochu* brewing because of the existence of *Rhizopus*, *Aspergillus*, *Saccharomyces cerevisiae*, and other amylolytic fungal species and yeasts [2]. However, the traditional starter (Qu) ferments spontaneously, and its microbial community formation is influenced by numerous variables, such as humidity, temperature, climate, and the microorganisms in the natural setting [3]. Consequently, a rise or fall in Qu standards affect the quality of rice *shochu* products directly. In recent decades, *shochu* manufacturers have typically chosen a pure-strain starter to initiate the brewing process because it enhances the stability of products [4,5,6,7,8]. Fungal molds and yeasts are essential microorganisms for rice *shochu* fermentation starters [2,9]. In the fermenting procedure of rice *shochu*, starch degradation and alcoholic fermentation are conducted simultaneously.

*Koji* is produced by solid culture using pure molds on cooked rice, which contains volatile flavor compounds and various hydrolase enzymes, such as glucoamylase, amylase, and protease [4,10]. The main genera of molds for rice wine are *Aspergillus* and *Rhizopus* [11,12,13]. The molds *Aspergillus kawachii*, *Aspergillus oryzae*, *Aspergillus Niger*, and *Rhizopus oryzae* are traditionally used in the rice wine brewing industry and have been widely accepted as safe to ingest [4,7,8].

The flavor of rice *shochu* is among the most significant factors that impact the product quality and consumer acceptance [14]. The volatile compounds in buckwheat, rice, and barley *shochu* were examined utilizing gas chromatography olfactometry (GC-O), aroma extract dilution analysis (AEDA), and GC-MS [15]. Flavor compounds present in young and aged *awamori shochu* were detected utilizing a three-stage volatile organic compound (VOC) concentration method, which consisted of on-line gas chromatography (GC) combined with a mass-selected detector (MSD) and a pulsed flame photometric detector (PFPD) [16]. The volatile components in various types of *shochu* were examined, and their characteristics and relationships were compared [5,17].

However, little attention has been paid to the effect of *koji* on the synthesis of FAAs and flavor substances in rice *shochu* manufacturing. Consequently, the purpose of this research was to examine the influences of different *koji* brewing starters on the volatile components and sensory attributes of rice *shochu*. Using high-performance liquid chromatography (HPLC) and headspace solid-phase microextraction–gas chromatography–mass spectrometry (HS-SPME-GC-MS), the FAAs and volatile substances in rice *shochu* were analyzed. Additionally, the sensory attributes of rice *shochu* were tested. Using the PLSR approach, the probable relationships among the FAAs, volatile flavor components, and sensory attributes of rice *shochu* were investigated more deeply. Eventually, this research aimed to develop a connection between the various types of *koji* brewing starters and the sensory quality of rice *shochu* generated by them, and to give a benchmark for determining the entry requirements for the production of suitable *koji*. The present study could provide a reference for rice *shochu* production using appropriate *koji* to enhance the flavor qualities.

## 2. Results and Discussion

### 2.1. Impacts of Koji on the FAAs in Rice Shochu 

The data listed in Table 1 show that Pro, Asp, Glu, Tyr, Arg, and Ala were the dominant amino acids in all the samples. This correlated with a previous study of cachaca, rum, and whisky amino acids, which observed higher content of Pro and Asp among samples [18].

The content of total amino acids in rice *shochu* using *koji* A-K and A-O was higher than in other samples, which were 30.586 ± 0.944 and 29.919 ± 0.278 mg/L, respectively. Significant increases (*p* < 0.05) in bitter and sour amino acids were observed in the rice *shochu* using the A-K starter. However, sweet FAAs in rice *shochu* using the R-O starter were drastically reduced (*p* < 0.05). Moreover, in the samples fermented for A-N and control *koji*, the concentrations of bitter amino acids were lower than that in the others (*p* < 0.05). These results indicated that *koji* made a significant contribution to the proportion of FAAs in rice *shochu*.

The major FAAs in the control samples were in the order of Asp > Pro > Glu > Tyr > Ala > His > Arg. In the A-K and A-O samples, the order changed to Pro > Asp > Glu > Tyr > Ala > His > Arg. In the A-N and R-O samples, the order changed to Pro > Asp > Glu > Arg > Ala > Tyr > His and Asp > Pro > Tyr > Glu > Arg > Ala > His, respectively. This suggested that *koji* altered the profiles of the major FAAs in rice *shochu*.

The degradation of the proteins from raw rice provided nitrogen for the growth of microbes during the fermentation process. FAAs were derived mainly from the strong acid protease breakdown of the protein in the rice material and the autolysis of microbes during fermentation [19]. In the distillation process, the amino acids that are concealed in a foam fog are introduced into the rice *shochu* [20]. FAAs elicited various tastes in rice *shochu*, such as bitter, sweet, and sour, and played an important role as an aroma compound in rice *shochu*. Thus, amino acids directly influence the taste of rice *shochu*.

### 2.2. Volatile Flavor Compounds in Rice shochu

Volatile flavor compounds were detected using GC-MS coupled with HS-SPME. There were altogether 66 volatile compounds in the rice *shochu*. In addition, 23 alcohols, 21 esters, 7 acids, 8 aldehydes, 4 ketones, and 3 other compounds were determined by mass spectra, pure reference compounds, and databases (Table 2). The amounts of flavor compounds in majority were significantly influenced by the different *koji*.

Alcohols were important flavor compounds and major components of rice *shochu* in this study. They had a non-negligible effect on the overall flavor of *shochu*. As listed in Table 2, the main alcohols detected in rice *shochu* were isoamyl alcohol, phenethyl alcohol, and isobutyl alcohol. These primarily produced distinctive floral, sweet, and rose aromas [21]. The phenethyl alcohol content in rice *shochu* using R-O *koji* was greatly increased compared with that using A-N *koji*. The isoamyl alcohol content in rice *shochu* using A-N *koji* was obviously reduced compared with that in the other samples. The phenethyl alcohol content in rice *shochu* using R-O *koji* was significantly increased compared with that of isoamyl alcohol. Alcohols might be formed either directly from sugar fermentation or indirectly through the catabolism of amino acids; hence, their amounts varied according to the distinct sugars and amino acids available [22]. Accordingly, it was highly likely that the actions of amylase, glucoamylase, and acid protease in koji were responsible for the majority of alcohols in rice *shochu*.

Esters were the second most quantifiable constituent of rice *shochu* (Table 2), and their extremely attractive fruity aroma had a significant effect on the attributes of rice *shochu*. The rice *shochu* made with *koji* A-K contained the greatest amount of esters, which was higher than that of A-O *koji* and A-N *koji*. Ethyl esters were the largest volatile flavor compounds in rice *shochu,* which were described as a “fruity, flower” aroma [21]. Phenylethyl acetate was higher in rice *shochu* fermented with A-K and A-O *koji* than that of A-N, R-O, and the control *koji*, which was described as a “rosy, honey” aroma [23].

During rice *shochu* fermentation, the production of esters by alcohol acetyl transferase employing acetyl-CoA and higher alcohols as substrates in microorganisms [24] was more important than the esterification of alcohols and fatty acids [25]. Consequently, the quantities of acetyl-CoA and higher alcohols as well as the activity of alcohol acetyl transferase [26] impacted ester production. The alcohol level was greater in A-O *shochu* than in A-N *shochu*. In addition, *Aspergillus oryzae* showed greater alcohol acetyl transferase action than *Aspergillus niger* [27]. This discovery partially addressed why the total amount of esters in A-O *shochu* was significantly greater than in A-N. The third most quantifiable constituent and flavor attribute of rice *shochu* was acids.

The outcomes showed that acetic acid and hexanoic acid were the most important volatile acids. Rice *shochu* prepared using R-O *koji* and the control generated the greatest amounts of hexanoic acid and acetic acid, respectively.

Carbonyl components, such as aldehydes and ketones, were studied in rice *shochu*. Acetaldehyde, acetal, phenylacetaldehyde, and decanal were detected in rice *shochu*, playing the major role of chemical transformation and oxidation in aldehyde generation. Moreover, 2,3-butanone was detected in A-O rice *shochu*, which had a greater impact on the aroma of the rice *shochu*, particularly the body’s softness and warmth [24].

Other components were also studied in rice *shochu*, such as phenols and 2-pentyl furan, which were available in modest quantities. Phenol originated from the debasement of ferulic acid, which is plentiful in grains [28].

*Koji* starters, owing to the effect of different microorganisms, effectively acted on rice *shochu* production. The change in *koji* significantly affected the content of volatile compounds in the rice *shochu*.

### 2.3. Analysis of the Ratio of Fusel Alcohols to Esters in Rice Shochu

**Figure 1:**
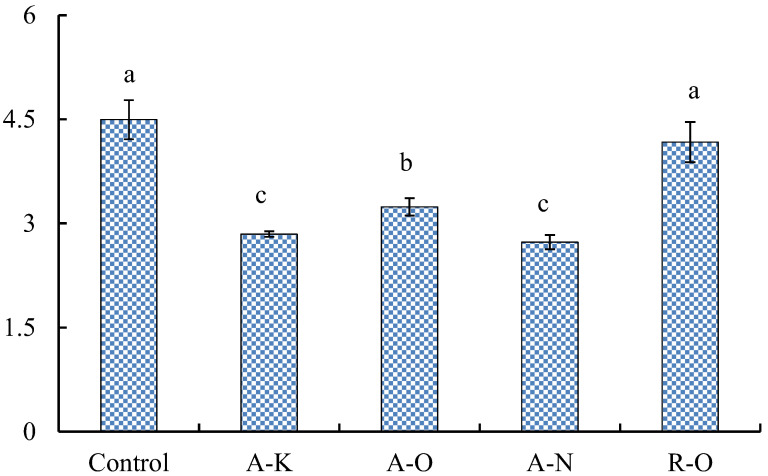
Various *koji* affect the proportion of fusel alcohols to esters in rice *shochu*. Means within different letters were significantly (*p* < 0.05) different.

Fusel alcohols, including 1-propanol, 1-butanol, 1-hexanol, 1-heptanol, 2,3-butanediol, 1-octanol, 1-nonanol, isobutyl alcohol, isoamyl alcohol, benzyl alcohol, and phenethyl alcohol, are manufactured from sugar fermentation directly or via catabolism from amino acids [22]. As the main flavor compound, fusel alcohols (FAs) are an essential factor that affected the quality of rice *shochu*. In this study, the ratio of FA/ester (FA/E) was used to estimate the characteristic in rice *shochu*. FA/E is a reliable index for evaluating the flavor distinction of distilled spirits [20,23]. The FA/E values of the rice *shochu* are shown in Figure 1. The FA/E in the control *shochu* was the highest at 4.495, whereas that in the A-K *shochu* was the lowest at 2.730. The FA/E ratio of the A-O *shochu* was 3.240. The intense alcoholic aroma in the control *shochu* could be attributed to the use of the control Qu. The control Qu had elevated nitrogen content in the fermenting mash, causing faster yeast growth and the synthesis of more fusel alcohols, hence resulting in a greater FA/E ratio relative to the other conditions. In contrast, the speed of yeast reproduction was lower in the primary stage of fermentation, which led to lower fusel alcohol content and reduced the ratio of FA/E.

### 2.4. Sensory Evaluation of Rice Shochu

**Figure 2:**
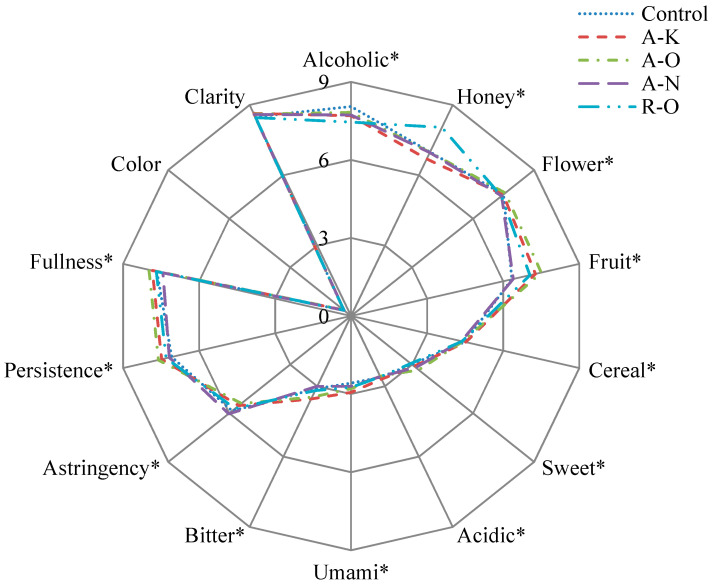
Sensory assessment of rice *shochu* for various *koji.* Within each sensory descriptor, * indicates significant variations from all *rice shochu* (*p* < 0.05).

Sensory evaluation was performed by evaluating the organoleptic quality. The mean sensory descriptor scores of rice *shochu* are shown in Figure 2. Statistical analysis showed that all descriptors except “color” and “clarity” demonstrated significant variables among the *shochu* samples (*p* < 0.05). Rice *shochu* using R-O *koji* presented the top score (8.05) with the “honey” descriptors among the five rice *shochu*, which could have been caused by the phenethyl alcohol in R-O *shochu*. All *shochu* samples exhibited similar intensity ratings for clarity. A-N *shochu* demonstrated higher scores with “astringency” descriptors than the others. The scores of “alcoholic” descriptors of control *shochu* were the highest, which could have significantly contributed to the higher FA/E. The “flower” and “fruit” descriptors in A-O *shochu* had a greater score than the other *shochu* samples, and “astringency” descriptors exhibited lower levels, which were considered to be the primary producers of several volatile components, such as isoamyl alcohol, phenethyl acetate, and isoamyl acetate. The quality of rice *shochu* brewed using *koji* with different characteristics varied significantly. Sensory analysis showed that the use of different *koji* exerted different effects on the flavor of rice *shochu*. In general, *shochu* fermented using A-O *koji* revealed a stronger aroma and more balanced flavor than *shochu* fermented using other *koji*.

### 2.5. Relationships among Volatile Compounds, FAAs, as Well as Sensory Qualities in Rice Shochu Made with a Variety of koji

**Figure 3:**
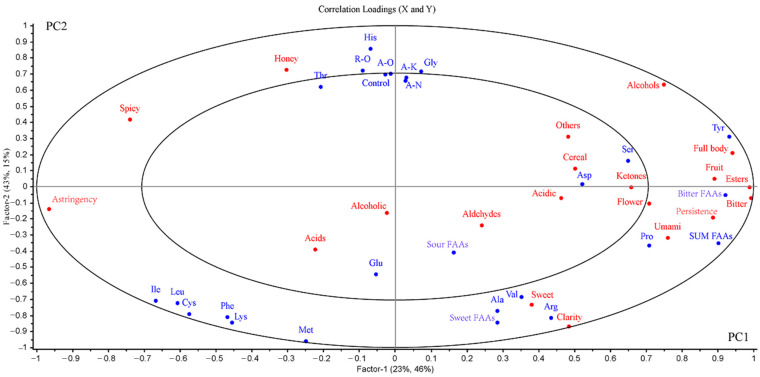
A brief overview of PLSR correlations loading plot of rice *shochu* for each individual *koji*. The X-matrix was composed of 5 rice *shochu* samples and FAAs, the Y-matrix was composed of the sensory properties and volatile compounds.

The purpose of PLSR was to examine the relationships between the FAAs, volatile compounds, and sensory features of rice *shochu* made with various *koji*. In Figure 3, the two large circles in the plot represent the 50% and 100% explained differences, respectively. While the fitting index of the model to the independent variable [R^2^(cum)] was 0.989, the prediction index of the model [Q^2^(cum)] was 0.983, and root-mean-square error (RMSE) < 0.001, which meant that the model was stable and reliable. In our research, the model was developed using five rice *shochu* samples (control, A-K, A-O, A-N, and R-O) and FAAs as the X-matrix and the sensory properties and volatile compounds as the Y-matrix. Meanwhile, the generated PLSR model contained two significant PC1 and PC2 components that explained 69% of the cross-validated variance of the X-matrix and 58% of the cross-validated variance of the Y-matrix, respectively (Figure 3).

As shown in Figure 3, *shochu* of the A-O, R-O, and control were located on the left side, which were distinguishable from the *shochu* of A-K and A-N positioned on the right side. *Shochu* of A-O, R-O, and the control showed honey, astringency, and spiciness, and were significantly positively associated with the sweet amino acids (e.g., Thr) and bitter amino acids (e.g., His). On the contrary, the alcohols, esters, phenols, as well as aldehydes found in *shochu* from the A-K and A-N regions were positively correlated with floral, sweet, full-bodied, and fruity flavors and the sweet amino acids (e.g., Gly and Ser). Based on the data presented, it is shown that *koji* starters, FAAs, and volatile compounds significantly influenced (*p* < 0.05) the rice *shochu*’s sensory features. Moreover, FAAs and volatile compounds are mainly responsible for the rice *shochu*’s characteristics.

## 3. Materials and Methods

### 3.1. Rice Koji Preparation

The molds *Aspergillus niger* CICC 2372 (A-N) and *Rhizopus oryzae* CICC 40260(R-O) were preserved in the China Center of Industrial Culture Collection, CICC; *Aspergillus kawachii* SICC 3.917(A-K) and *Aspergillus oryzae* SICC 3.79 (A-O) were preserved in the Sichuan Center of Industrial Culture Collection, SICC. For 48 h at 28 °C, the molds were grown on slants of activated potato dextrose agar (PDA) (potato 200 g/L, glucose 20 g/L, and agar 20 g/L; Beijing Aoboxing Biological Co., Ltd., Beijing, China). Slants were placed in a 500 mL Erlenmeyer flask with sterilized spore-producing medium [1]. Following 72 h of cultivation at 28 °C, 100 mL of sterile water was poured to suspend the spores, and the suspension was filtered through sterile gauze. The filtrate including spores was utilized to make rice *koji*. The culture of rice *koji* was formed by piling up and cultivating in an incubator (model HMJ-II-300, Shanghai Yuejin Medical Instrument Co., Ltd., Shanghai, China) at 38 °C and 95% relative humidity for 48 h; then, at 32 °C, without changing the relative humidity, it was incubated for a further 24 h, and it was then incubated at 40 °C and 50% relative humidity for 12 h finally [29].

### 3.2. Preparation of Yeast

*Saccharomyces cerevisiae* CICC 1050 was preserved in the China Center of Industrial Culture Collection, CICC. After 48 h of growth at 28 °C, a slant inoculation of CICC 1050 strain was transferred to a 50 mL Erlenmeyer flask containing 30 mL of sterile *koji* extract media. After 72 h of stationary cultivation at 25 °C and a hemocytometer count of 3 × 10^8^ cells/mL, the cells were available for additional usage [30]. *Koji* extract medium was prepared by dipping 200 g of *koji* into 700 mL of water, incubating it at 60 °C for 12–16 h, and filtering it through filter paper to remove suspended solids. The filtered solution was adjusted to 10° Brix with water before use [31].

### 3.3. Preparation of Rice Shochu

**Figure 4:**
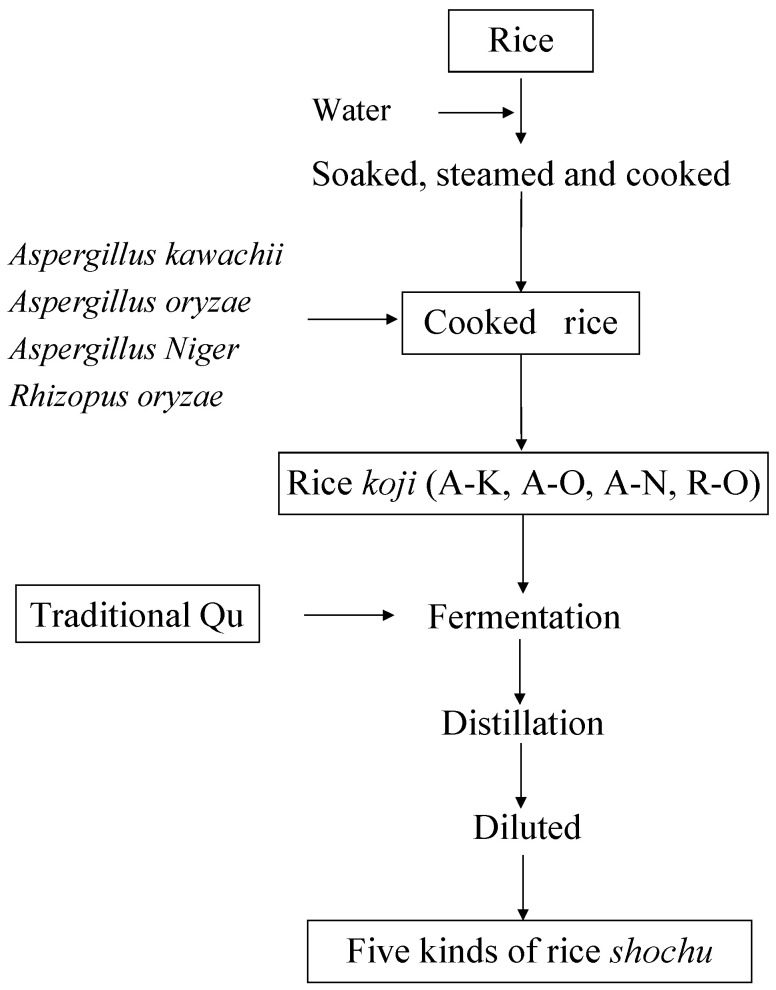
Research as well as production of rice *shochu* by *koji* as depicted by a flowchart.

The main preparation process for rice *shochu* is shown in Figure 4. The initial stage of fermentation was conducted at 25 °C for 3 d in a stationary manner by supplying 1 mL of yeast inoculum and 100 mL sterile water and 82.5 g rice *koji* produced by each specific mold (A-K, A-O, A-N, and R-O). The second step of fermentation was started by adding cooked rice (rice weight 250 g) and sterile water in a volume of 450 mL to the flask at 25 °C for 16 d with daily shaking by hand.

Using the method originally adopted by our research group [1], the fermented mash was distilled by a rotary evaporator (N-1300; Tokyo Eyela Co., Ltd., Tokyo, Japan) with a pressure controller (NVC-3000; Tokyo Eyela Co., Ltd.). Then, 40% of the fermented mash was distilled before the distillation process ceased. The distillate was then mixed with deionized water to reach a final ethanol concentration of 25% (*v*/*v*) as rice *shochu*.

Brewing of rice *shochu* was divided into 4 groups according to the rice *koji* used (Figure 4). The traditional starter Qu for rice wine from Yibin Sichuan was used as the control. A schematic of the investigation and development of rice *shochu* using the *koji* starter fermentation of rice is shown in Figure 4.

### 3.4. FAA Analysis

The rice *shochu* samples were filtered via a 0.22 μm filter membrane. The filtrate was used for further investigation. The content of FAAs in the samples was assessed via high-performance liquid chromatography (HPLC) utilizing an Agilent HPLC system 1100 equipped with an Agilent C18 column (250 mm × 4.6 mm × 5 μm), in accordance with the previous method, with minor adjustments [32,33]. Specifically, mobile phase A (pH 7.2): 27.6 mmol/L sodium acetate–riethylamine–tetrahydro furan (500:0.11:2.5); mobile phase B (pH 7.2): 80.9 mmol/L sodium acetate–methanol–acetonitrile (1:2:2); elution procedure: 0 min, 8% B; 17 min, 50% B; 20.1 min, 100% B; 24 min, 0% B; flow rate: 1.0 mL/min; column temperature: 40 °C; detection wavelength of ultraviolet detector (VWD): 338 nm; detection wavelength of proline: 262 nm.

### 3.5. Extraction and Quantification of Volatile Flavor Compounds

The volatile flavor component concentration of *shochu* samples was determined using solid-phase microextraction (SPME) and GC-MS in compliance with a previous method, with slight modifications [34,35]. Rice *shochu* samples were diluted to 10% ethanol with boiled ultrapure water. In a 15 mL headspace container, 1.2 g of NaCl and 10 µL of 2-octanol (54.827 mg/L in absolute ethanol) were introduced to samples of diluted rice *shochu* (3 mL). An SPME device and 50/30 μm (DVB/CAR/PDMS)-coated fibers (Supelco Inc., Bellefonte, PA, USA) were employed. The samples were evenly balanced at 60 °C for 15 min, recovered for 45 min, and then the fiber was injected into the injection port of the GC (250 °C) for 5 min to desorb the analytes. A gas chromatograph with a connected mass spectrometer (QP2020, Shimadzu Co., Ltd., Kyoto, Japan) was employed with a HP-INNOWAX column (60 m × 0.25 mm × 0.25 μm, Agilent Technologies, Inc., Santa Clara, CA, USA). The gas chromatography oven temperature was first held at 40 °C for 5 min, and then increased at a rate of 5 °C/min to 100 °C and 6 °C/min to 230 °C (held for 10 min). The carrier gas was helium at a flow rate of 1.0 mL/min (99.999%). The injection was carried out via splitless mode. At 70 eV, the ion source temperature was 230 °C, and the electron impact (EI) mode mass detector was applied. By measuring the total ion currents in the *m*/*z* 10–400 mass range, chromatograms were created. The volatile substances were validated by contrasting the spectra with the results of a mass spectrum library search (NIST14s), pure reference compounds, and retention indices (RIs) related to the literature database. Using 2-octanol as an internal standard, semi-quantitation of the volatile compounds was achieved [3].

### 3.6. Sensory Evaluation of the Rice Shochu

Sensory assessment of the rice *shochu* samples was carried out by a well-trained team of 10 judges (5 females and 5 males between 20 and 50 years old, i.e., *shochu* critic, professional *shochu* technicians) as in the previously detailed method, similar to rice wine, with minor adjustments [36]. The judges were trained in compliance with [37]. The 14 sensory attributes of rice *shochu* were identified to be aroma (alcohol, honey, flower, fruit, and cereal), taste (sweet, acidic, umami, astringency, and bitter), mouthfeel (persistence and fullness), and appearance (color and clarity). The intensity ratings of descriptors were scored from 0 to 9 (0: none; 1–2: very weak; 3–4: ordinary; 5–6: moderate; 7–8: intense; 9, very intense) [38,39,40]. Standards for defining the sensory descriptors were part of the training and taste examinations. As per the earlier publications [41,42], sensory assessment was performed in a sensorial testing room at 20 °C with a uniform light source and in the absence of sound and any disturbing stimuli. The blind examination of these samples was carried out utilizing randomly selected contents, vigorous whirling, and inhaling of the headspace vapor. The rice *shochu* samples (30 mL) were placed in the same cup and provided in coded form for rating at 20 °C. Throughout the test, water was utilized to rinse the mouth. A card presenting all sensory attributes with definitions and nine-point scales (from 0–9) was used for the *shochu* tasting test. Judges were asked to quantify each sensory descriptor of the *shochu* samples. The study was reviewed and approved by the Yibin University IRB and informed consent was obtained from each subject prior to their participation in the study.

### 3.7. Statistical Analysis

All analyses were performed in triplicate. Results were examined by one-way analysis of variance (ANOVA) employing SPSS (version 22.0, IBM Corp., Armonk, NY, USA). Using the Duncan test at *p* < 0.05, significant variations across the data were found. The findings were presented as mean ± standard deviation (SD). The possible relationships between FAAs, volatile compounds, sensory features, and *koji* were investigated utilizing PLSR and Unscrambler (version 9.7, CAMO ASA, Oslo, Norway). Using complete cross-validation, all regression models were validated.

## 4. Conclusions

In this study, the FAAs, flavor compounds, and sensory qualities of rice *shochu* fermented with A-K, A-O, A-N, and R-O *koji* were examined. The results showed that rice *shochu* containing A-O and A-K *koji* contained higher levels of FAAs and aroma compounds. The major proportions of aroma components in rice *shochu* made with A-O and A-K *koji* were similar to those in the traditional Qu (control), although the most notable changes arose from the relative abundance of alcohols and esters. Sensory evaluation demonstrated that A-O *shochu* exhibited high levels of “flower” and “fruit”, whereas the control *shochu* was intensely “alcoholic”. Rice *shochu* exhibited a similar intensity rating for clarity. PLSR analysis revealed that the type of *koji*, FAAs, and flavor compounds had a notable effect on the sensory properties of rice *shochu*. The manufacturing of rice *shochu* with a variety of *koji* is a viable solution for controlling the features of rice *shochu*.

Practical Application: The effects of different *koji* brewing starters on the volatile compounds and sensory quality of rice *shochu* were explored. Then, this study sought to establish a correlation between the different types of *koji* brewing starters and the sensory quality of rice *shochu* produced by them, and ultimately provide a reference for establishing the selection criteria for the development of appropriate *koji*. Our findings would provide a reference for rice *shochu* production using *koji* as a saccharification and liquefaction starter.

## Figures and Tables

**Table 1:** The impacts of FAAs in rice *shochu* using different *koji* (mg/L).

	Control	A-K	A-O	A-N	R-O
Sweet amino acids					
Gly	0.930 ± 0.067 ^a^	0.363 ± 0.016 ^b^	0.986 ± 0.042 ^a^	0.406 ± 0.027 ^b^	0.907 ± 0.056 ^a^
Ala	2.282 ± 0.148 ^a^	2.413 ± 0.184 ^a^	2.408 ± 0.132 ^a^	2.362 ± 0.146 ^a^	1.686 ± 0.027 ^b^
Ser	0.364 ± 0.027 ^b^	0.320 ± 0.010 ^c^	0.446 ± 0.011 ^a^	0.250 ± 0.017 ^d^	0.290 ± 0.017 ^c^
Thr	0.496 ± 0.028 ^a^	0.069 ± 0.005 ^c^	0.497 ± 0.038 ^a^	0.255 ± 0.015 ^b^	0.473 ± 0.020 ^a^
Pro	6.364 ± 0.444 ^b^	7.207 ± 0.630 ^a^	6.480 ± 0.054 ^ab^	6.299 ± 0.206 ^b^	6.155 ± 0.418 ^b^
Lys	0.491 ± 0.024 ^d^	0.839 ± 0.064 ^b^	0.625 ± 0.040 ^c^	1.547 ± 0.063 ^a^	0.539 ± 0.035 ^d^
Cys	0.020 ± 0.001 ^c^	0.059 ± 0.003 ^b^	0.017 ± 0.002 ^c^	0.241 ± 0.020 ^a^	0.008 ± 0.001 ^c^
∑	10.947 ± 0.428 ^a^	11.271 ± 0.612 ^a^	11.485 ± 0.120 ^a^	11.360 ± 0.256 ^a^	10.05 8± 0.312 ^b^
Bitter amino acids					
Val	0.070 ± 0.002 ^d^	0.237 ± 0.009 ^b^	0.269 ± 0.021 ^a^	0.257 ± 0.009 ^ab^	0.181 ± 0.014 ^c^
Leu	0.233 ± 0.013 ^b^	0.246 ± 0.018 ^b^	0.226 ± 0.013 ^b^	0.290 ± 0.017 ^a^	0.234 ± 0.007 ^b^
Ile	0.112 ± 0.010 ^b^	0.118 ± 0.023 ^b^	0.108 ± 0.008 ^b^	0.202 ± 0.077 ^a^	0.113 ± 0.005 ^b^
Met	0.018 ± 0.001 ^c^	0.058 ± 0.006 ^b^	0.056 ± 0.011 ^b^	0.126 ± 0.007 ^a^	0.006 ± 0.001 ^d^
Phe	0.082 ± 0.008 ^d^	0.139 ± 0.016 ^b^	0.128 ± 0.011 ^bc^	0.281 ± 0.017 ^a^	0.113 ± 0.004 ^c^
Arg	1.159 ± 0.075 ^c^	2.992 ± 0.269 ^a^	2.905 ± 0.000 ^a^	2.863 ± 0.095 ^a^	1.734 ± 0.106 ^b^
His	1.371 ± 0.079 ^a^	1.013 ± 0.070 ^b^	0.407 ± 0.028 ^c^	0.372 ± 0.023 ^c^	1.399 ± 0.051 ^a^
Tyr	2.626 ± 0.183 ^c^	4.339 ± 0.047 ^a^	4.395 ± 0.275 ^a^	1.538 ± 0.076 ^d^	3.721 ± 0.244 ^b^
∑	5.669 ± 0.308 ^d^	9.142 ± 0.335 ^a^	8.495 ± 0.273 ^b^	5.927 ± 0.203 ^d^	7.501 ± 0.255 ^c^
Sour amino acids					
Asp	6.412 ± 0.053 ^a^	6.533 ± 0.053 ^a^	6.410 ± 0.009 ^a^	6.414 ± 0.056 ^a^	6.460 ± 0.105 ^a^
Glu	3.412 ± 0.144 ^b^	3.639 ± 0.057 ^a^	3.529 ± 0.065 ^ab^	3.6778 ± 0.085 ^a^	3.602 ± 0.072 ^a^
∑	9.824 ± 0.162 ^c^	10.173 ± 0.172 ^a^	9.939 ± 0.057 ^bc^	10.092 ± 0.029 ^ab^	10.062 ± 0.093 ^ab^
SUM	26.440 ± 0.729 ^b^	30.586 ± 0.944 ^a^	29.919 ± 0.278 ^a^	27.380 ± 0.469 ^b^	27.621 ± 0.487 ^b^

Note: values with distinct lowercase characters within the same row were statistically significant (*p* < 0.05). All findings are shown as the mean ± standard deviation (*n* = 3).

**Table 2:** Relative changes in the amounts of volatile substances in rice *shochu* using different *koji*.

Compound		Multiple of Variation			
	Control	A-K	A-O	A-N	R-O
Alcohols					
1-Propanol	1.0	2.1	1.9	0.7	1.7
Isobutyl alcohol	1.0	1.4	1.4	0.5	1.0
1-Butanol	1.0	3.7	3.1	0.8	3.5
Isoamyl alcohol	1.0	0.9	1.0	0.4	0.8
1-Hexanol	1.0	3.5	2.5	nd	0.5
1-Heptanol	1.0	8.3	4.4	4.8	7.2
2,3-Butanediol	1.0	3.8	7.9	1.8	5.6
1-Octanol	1.0	25.8	15.8	3.9	15.6
1-Nonanol	nd	nd	+	+	+
3-Methylmercapto propanol	1.0	2.0	2.2	0.9	2.4
Citronellol	1.0	2.8	nd	0.8	nd
Benzyl alcohol	1.0	0.5	0.3	0.3	1.1
Phenethyl Alcohol	1.0	0.9	1.0	0.4	1.1
1-Dodecanol	1.0	14.7	11.1	9.8	11.7
Epicubenol	1.0	1.9	5.7	0.8	6.8
Cedrol	1.0	25.1	24.9	26.0	63.9
Ylangenol	nd	+	+	+	nd
1-Hexadecanol	1.0	1.4	nd	1.1	nd
Nerolidol	1.0	1.5	0.6	nd	nd
α-Cadinol	1.0	2.6	3.3	1.1	4.9
Spathulenol	1.0	nd	nd	nd	3.2
α-Santalol	1.0	58.1	2.1	nd	17.0
α-Bisabolol	1.0	2.8	3.0	nd	6.3
∑	1.0	1.0	17	0.4	1.0
Esters					
Ethyl Acetate	1.0	1.3	1.3	0.7	1.0
Ethyl 2-methylpropionate	1.0	0.8	1.1	0.9	0.9
Ethyl caproate	1.0	9.6	11.3	10.5	6.6
Ethyl heptanoate	1.0	1.1	0.6	0.5	0.6
Ethyl lactate	1.0	0.3	0.2	0.8	0.2
Isoamyl acetate	1.0	1.0	1.9	0.4	0.6
Ethyl caprylate	1.0	1.1	1.2	0.5	1.0
Ethyl 2-hydroxy-4-methylvalerate	1.0	16.4	16.0	nd	10.4
Ethyl nonanoate	1.0	18.7	16.9	11.6	13.8
Butyrolactone	1.0	nd	6.0	nd	nd
Ethyl decanoate	nd	+	+	+	+
Ethyl benzoate	1.0	2.1	1.1	0.2	1.7
Diethyl succinate	1.0	1.5	1.4	0.4	1.1
Ethyl phenylacetate	1.0	2.1	1.7	1.1	1.2
Phenethyl acetate	1.0	1.0	1.9	0.7	1.0
Ethyl myristate	1.0	38.1	37.0	nd	26.7
Ethyl palmitate	1.0	9.0	1.5	nd	1.6
Methyl stearate	1.0	0.6	0.6	0.2	nd
Ethyl octadecanoate	1.0	3.4	2.9	nd	3.3
Ethyl oleate	1.0	8.2	7.7	2.8	7.3
Ethyl linoleate	1.0	7.0	6.7	3.3	2.3
∑	1.0	1.6	1.5	0.7	1.1
Acids					
Acetic acid	1.0	0.1	0.0	0.5	0.1
Hexanoic acid	1.0	1.1	1.8	1.7	2.0
Myristic acid	nd	nd	+	nd	nd
Octanoic acid	1.0	69.6	173.4	140.0	151.0
Nonanoic acid	1.0	nd	nd	0.5	nd
n-Decanoic acid	1.0	198.0	15.0	26.7	nd
n-Hexadecanoic acid	nd	nd	+	nd	+
∑	1.0	1.2	1.4	1.5	1.4
Aldehydes					
Acetaldehyde	1.0	1.6	1.7	1.0	1.6
Acetal	1.0	1.0	1.2	1.0	1.0
Hexanal	nd	nd	+	nd	nd
Nonanal	1.0	0.7	0.5	3.1	0.5
Furfural	1.0	nd	2.1	1.5	11.8
Decanal	1.0	0.7	0.5	3.1	0.6
Benzaldehyde	1.0	nd	nd	0.5	nd
Benzeneacetaldehyde	1.0	1.4	1.3	1.3	1.2
∑	1.0	1.2	1.3	1.3	1.3
Ketones					
2,3-Butanone	nd	nd	+	nd	nd
2-Octanone	1.0	1.6	2.4	nd	2.0
3-Nonanone	nd	+	+	+	nd
Acetoin	1.0	nd	nd	+	+
∑	1.0	10	8.9	0.6	2.5
Others					
2-Pentyl furan	1.0	2.5	26.1	nd	18.9
Naphthalene	nd	+	+	nd	nd
Phenol	nd	nd	nd	+	nd
∑	1.0	3.2	27.8	0.6	18.9
SUM	1.0	1.2	1.2	0.5	1.0

Note: nd, not detected. Take the control group as the baseline and compare the treatment as a multiple of increase or decrease. “+” indicates that a certain component was not detected in the control group but was detected in the experimental group, indicating an increase in content.

## Data Availability

The data that support the findings of this study are available from the corresponding author upon reasonable request.
